# Production of C2–C4 diols from renewable bioresources: new metabolic pathways and metabolic engineering strategies

**DOI:** 10.1186/s13068-017-0992-9

**Published:** 2017-12-13

**Authors:** Ye Zhang, Dehua Liu, Zhen Chen

**Affiliations:** 1 Department of Chemical Engineering, Tsinghua University, Beijing, 100084 China; 2 Key Lab of Industrial Biocatalysis, Ministry of Education, Tsinghua University, Beijing, 100084 China; 3 Tsinghua Innovation Center in Dongguan, Dongguan, 523808 China; 4 Center of Synthetic and Systems Biology, Tsinghua University, Beijing, 100084 China

**Keywords:** Diols, Bioresource, Pathway design, New metabolic pathway, Metabolic engineering

## Abstract

C2–C4 diols classically derived from fossil resource are very important bulk chemicals which have been used in a wide range of areas, including solvents, fuels, polymers, cosmetics, and pharmaceuticals. Production of C2–C4 diols from renewable resources has received significant interest in consideration of the reducing fossil resource and the increasing environmental issues. While bioproduction of certain diols like 1,3-propanediol has been commercialized in recent years, biosynthesis of many other important C2–C4 diol isomers is highly challenging due to the lack of natural synthesis pathways. Recent advances in synthetic biology have enabled the de novo design of completely new pathways to non-natural molecules from renewable feedstocks. In this study, we review recent advances in bioproduction of C2–C4 diols, focusing on new metabolic pathways and metabolic engineering strategies being developed. We also discuss the challenges and future trends toward the development of economically competitive processes for bio-based diol production.

## Background

Diols are compounds containing two hydroxyl groups. C2–C4 diols (ethylene glycol, propanediols, and butanediols) are very important platform chemicals which have been widely used in various areas including solvents, fuels, polymers, cosmetics, and pharmaceuticals [1, 2]. For example, ethylene glycol (EG), 1,3-propanediol (1,3-PDO), and 1,4-butanediol (1,4-BDO) are among the most important building blocks of polymer industry for the synthesis of polyethylene terephthalate (PET), polypropylene terephthalate (PTT), and polybutylene terephthalate (PBT) [3, 4]. Annually, more than 18 million tons of C2–C4 diols are produced from chemical processes using fossil resource as the starting raw material [5, 6]. Owing to the diminishing fossil resource, fluctuation of oil prices, and increasing concerns about environmental problems, production of C2–C4 diols from renewable bioresources by environmentally benign biological processes has received significant interest [7, 8].

**Fig. 1:**
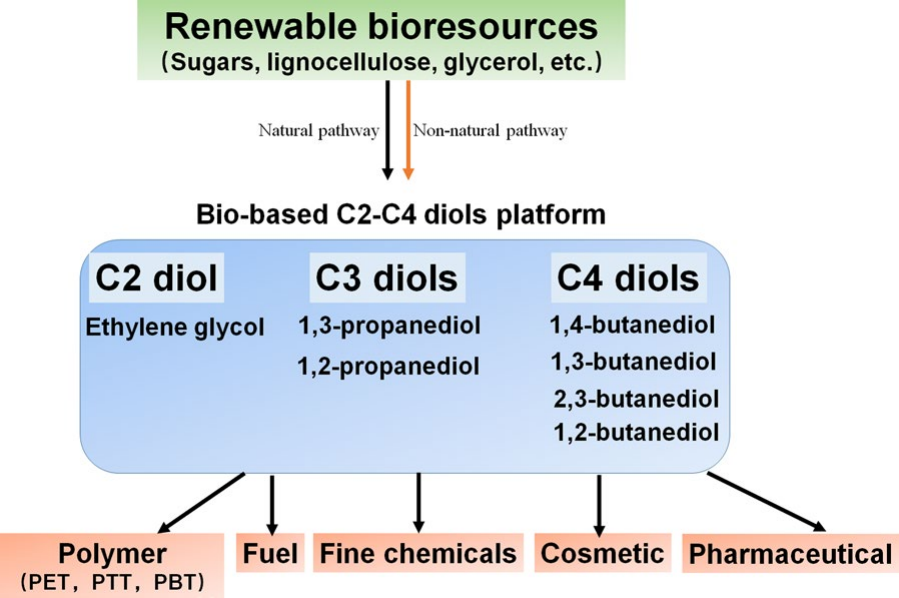
Isomers of C2–C4 diols and their application. *PET* polyethylene terephthalate, *PTT* polypropylene terephthalate, *PBT* polybutylene terephthalate

There exist different isomers of C2–C4 diols, including the following: EG of C2 diol; 1,3-PDO and 1,2-propanediol (1,2-PDO) of C3 diols; 1,4-BDO, 1,3-butanediol (1,3-BDO), 1,2-butanediol (1,2-BDO), and 2,3-butanediol (2,3-BDO) of C4 diols (Fig. 1). Production of all members of C2–C4 diols by biological approaches is highly challenging due to the lack of natural synthesis pathways for some isomers. There are intensive studies to modify natural pathways to produce natural C3 and C4 diols, namely 1,2-PDO, 1,3-PDO, and 2,3-BDO [9–15]. Especially, biological production of 1,3-PDO and 2,3-BDO has reached high titers and yields enough for commercial-scale manufacture [16, 17]. Dupont has successfully commercialized the bio-based 1,3-PDO in the early 2000s, which has become a milestone in metabolic engineering [17]. However, natural synthesis pathways to molecules like EG, 1,4-BDO, 1,3-BDO, and 1,2-BDO from cheap substrates have not been previously known. Production of these non-natural molecules via biological routes toward industrial application is much more complicated than natural ones. It generally requires four steps, including (a) in silico design and selection of non-natural metabolic pathways; (b) screening and engineering of biological parts, especially novel enzymes, to catalyze the desired chemical reactions; (c) assembling different biological parts and balancing metabolic pathways; and (d) systems metabolic engineering of chassis [18–21]. With these efforts, Genomatica has recently demonstrated the successful commercialization of bio-based 1,4-BDO, a typical non-natural molecule [22, 23]. These strategies can also be used to design novel or better pathways to produce natural molecules.

Prediction of new or non-natural pathways is often the first step toward bioproduction of non-natural molecules. With the recent development of synthetic biology and bioinformatics, different algorithms and prediction tools have been developed to aid in in silico pathway design and metabolic engineering [24–26]. Especially, several pathway prediction tools, such as BNICE and RetroPath which consider also promiscuous enzyme reactions, can be used for prediction and prioritization of various possible pathways to a given compound [27, 28]. In these computational frameworks, a chemical compound is generally coded into a molecular signature containing the information about atom composition and atom connection. Enzyme reactions are represented by a series of reaction rules by subtracting the substrates’ signatures from the products’ signatures. Based on a list of reactants and these general reaction rules, BNICE generates new pathways and molecules iteratively, which can be used to significantly extend currently known metabolic space [27]. On the other hand, RetroPath uses retrosynthesis algorithms to perform a backward search for potential routes to a targeted product through the iterative application of a defined set of biochemical transformation rules [28]. All of the potential pathways can then be ranked with the consideration of pathway thermodynamics, pathway length, the toxicity of intermediates, maximum yield, etc. [24, 25]. Several recently developed computational tools can also integrate the predicted new pathways into a specific genome-scale metabolic network, guiding further optimization process of the selected host with the combination of constraint-based flux analysis tools [26, 29].

Although different non-natural metabolic pathways can be designed, construction and optimization of non-natural pathways and systems metabolic engineering of chassis to increase product titers, yields, and productivities are still the limiting steps [30–34]. In this review, recent efforts to develop industrial strains for C2–C4 diols are summarized. Especially, we focus on new metabolic pathways and metabolic engineering strategies developed in recent years to produce C2–C4 diols from a variety of renewable bioresources. We also figure out the challenges and future trends toward the development of economically competitive bioprocesses for these diols.

## Metabolic engineering for the production of C2 diol: ethylene glycol

**Fig. 2:**
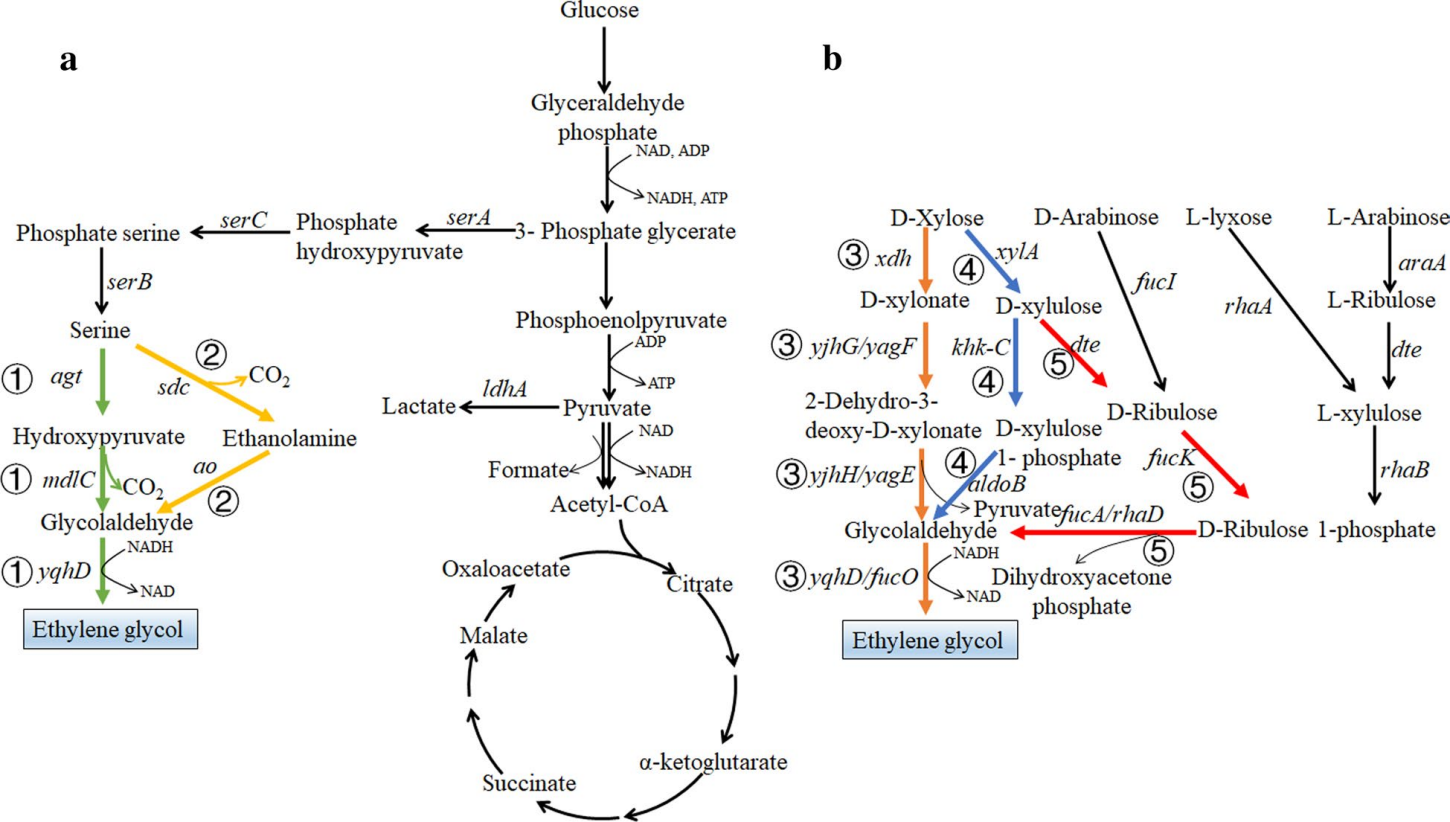
Metabolic pathways for the production of ethylene glycol (EG). **a** Production of EG from glucose by serine-derived metabolic pathways. Two metabolic pathways have been proposed to convert serine into EG: ① (illustrated by green colors) serine dehydrogenase or transaminase (*agt*), α-keto acid decarboxylase (*mdlC*), and alcohol dehydrogenase (*yqhD*); ② (illustrated by yellow colors) serine decarboxylase (*sdc*), monoamine oxidase (*ao*), and alcohol dehydrogenase (*yqhD*) as route ①. **b** Production of EG from pentoses by different metabolic pathways. d-Xylose can be converted into EG by three proposed pathways: ③ (illustrated by orange colors) d-xylose dehydrogenase (*xdh*), d-xylonate dehydratase (*yjhG/yagF*), 2-dehydro-3-deoxy-d-xylonate aldolase (*yjhH/yagE*), and alcohol dehydrogenase (*yqhD/fucO*); ④ (illustrated by blue colors) xylose isomerase (*xylA*), hexokinase (*khk*-*C*), aldolase (*aldoB*), and alcohol dehydrogenase (*yqhD/fucO*) as route ③; ⑤ (illustrated by red colors) xylose isomerase (*xylA*) as route ④, d-tagatose epimerase (dte), fuculokinase (*fucK*), d-ribulose 1-phosphate aldolase (*fucA/rhaD*), and alcohol dehydrogenase (*yqhD/fucO*) as route ③. d-Arabinose can be converted into EG by l-fucose isomerase (*fucI*), fuculokinase (*fucK*), d-ribulose 1-phosphate aldolase (*fucA/rhaD*), and alcohol dehydrogenase (*yqhD/fucO*). l-Lyxose can be converted into EG by l-rhamnose isomerase (*rhaA*), l-rhamnulokinase (*rhaB*), d-ribulose 1-phosphate aldolase (*fucA/rhaD*), and alcohol dehydrogenase (*yqhD/fucO*). l-Arabinose can be converted into EG by l-arabinose isomerase (*araA*), d-tagatose epimerase (DTE), l-rhamnulokinase (*rhaB*), d-ribulose 1-phosphate aldolase (*fucA/rhaD*), and alcohol dehydrogenase (*yqhD/fucO*)

EG is a highly important commodity chemical with an annual production of about 15 million tons [5]. It is primarily used as an antifreeze and a monomer for synthesizing over 25 million tons of PET [7]. Currently, EG is mainly produced by chemical routes using ethylene derived from petrochemical industry. Some recent efforts have tried to use bio-based ethanol to produce ethylene, which is then converted into EG by chemical processes [5]. Although this EG is derived from bioresources, the whole production process is energy intensive and cost ineffective. Thus, there is a great need and good opportunity to develop efficient bioprocesses to directly produce EG from renewable bioresources. There are no known natural pathways to directly synthesize EG from sugars. Based on partially known d-xylonate catabolism and in silico analysis using the metabolite prediction system of UM-BBD (University of Minnesota Biocatalysis and Biodegradation Database), Liu et al. have proposed a d-xylonate-dependent pathway (modified Dahms pathway) and demonstrated the first example of EG biosynthesis from pentose xylose by engineered *Escherichia coli* [35]. This metabolic pathway consists of four steps: first, xylose is oxidized to d-xylonate by d-xylose dehydrogenase; d-xylonate is then converted to 2-dehydro-3-deoxy-d-xylonate by d-xylonate dehydratase; 2-dehydro-3-deoxy-d-xylonate is cleaved into glycolaldehyde and pyruvate by a keto acid aldolase; finally, glycolaldehyde is converted to EG by alcohol dehydrogenase (route ③ of Fig. 2b). In *E. coli*, there are two native d-xylonate dehydratases encoded by genes *yjhG* and *yagF* and two 2-dehydro-3-deoxy-d-xylonate aldolases encoded by genes *yjhH* and *yagE*, which can catalyze the conversion of d-xylonate to glycolaldehyde [36]. There are also different inherent alcohol dehydrogenases, such as NADPH-dependent aldehyde reductase YqhD and NADH-dependent aldehyde reductase FucO, which can efficiently catalyze the reduction of glycolaldehyde to EG [37]. Thus, a recombinant *E. coli* can produce about 0.4 g/L EG with the introduction of only a heterologous *xdh* gene encoding d-xylose dehydrogenase from *Caulobacter crescentus* [35]. To further increase the production of EG, different strategies have been tried, including (1) deletion of *xylA* gene encoding xylose isomerase to reduce xylose isomerization; (2) overexpression of *yqhD* gene to enhance the reduction of glycolaldehyde; (3) and deletion of *aldA* gene encoding lactaldehyde dehydrogenase to reduce the oxidation of glycolaldehyde to glycolate [35]. The first two strategies were shown to be effective to increase the production of EG, and the engineered strain with both modifications achieved 11.7 g/L EG at a yield of 0.29 g/g xylose and a productivity of 0.24 g/L/h in batch fermentation within a controlled bioreactor (37 °C, pH 7.0, 350 rpm). Deletion of *aldA* gene to abolish glycolate accumulation, however, resulted in a significant accumulation of toxic intermediate d-xylonate and growth inhibition. Thus, metabolic pathway balancing should be further carried out by fine-tuning expression of the *xdh* gene and genes encoding downstream enzymes (e.g., *yjhG* and *yagF*) [38].

Besides d-xylonate-dependent pathway, another two different pathways to convert xylose to EG have also been proposed recently (Fig. 2b). In a d-xylulose 1-phosphate-dependent pathway (route ④ of Fig. 2b), xylose is first isomerized to d-xylulose by xylose isomerase [39]. d-Xylulose is phosphorylated to d-xylulose 1-phosphate by d-xylulose-1 kinase, which is further cleaved into glycolaldehyde and dihydroxyacetone phosphate by d-xylulose 1-phosphate aldolase. Glycolaldehyde is then converted to EG by the same alcohol dehydrogenase as route ③. Alkim et al. have implemented this pathway in *E. coli* with the introduction of human hexokinase encoded by the *khk*-*C* gene and aldolase encoded by the *aldoB* gene [39]. Blocking xylose consumption pathways via the deletion of *xylB* (d-xylose dehydrogenase) and *aldA* genes, and overexpression of the inherent NADH-dependent alcohol dehydrogenase gene *fucO* can significantly improve the production of EG. The engineered strain can produce 20 g/L EG with a yield of 0.38 g/g xylose and a productivity of 0.37 g/L/h in batch fermentation within a controlled bioreactor (37 °C, pH 7.0, dissolved oxygen over 40%) [39]. Different from the study of Liu et al. [35], deletion of *aldA* gene abolished the synthesis of glycolate without obvious accumulation of d-xylonate in this engineered strain, which contributed to the higher yield and titer of EG.

Similar to d-xylulose 1-phosphate-dependent pathway, a d-ribulose 1-phosphate-dependent pathway was proposed to covert d-xylose (route ⑤ of Fig. 2b) and other pentoses (d-arabinose, l-lyxose, and l-arabinose) to EG (Fig. 2b) [6]. In this pathway, d-xylulose derived from xylose isomerization is epimerized to d-ribulose by d-tagatose epimerase (*dte*). d-Ribulose is phosphorylated to d-ribulose 1-phosphate by fuculokinase (*fucK*), which is further cleaved into glycolaldehyde and dihydroxyacetone phosphate by d-ribulose 1-phosphate aldolase (*fucA* or *rhaD*). Glycolaldehyde is then converted to EG by the same alcohol dehydrogenase as other mentioned pathways. *E. coli* bears inherent fuculokinase and d-ribulose 1-phosphate aldolase. Thus, with the expression of only a heterologous *dte* gene from *Pseudomonas cichorii* and deletion of *xylB* and *aldA* genes, the strain can already accumulate EG using xylose as a substrate. Overexpression of *fucK*, *fucO*, and *fucA* genes in low-copy plasmid further improved the performance of the strain, which can produce 40 g/L EG with a yield of 0.35 g/g xylose and a productivity of 0.58 g/L/h in fed-batch fermentation within a controlled bioreactor (37 °C, pH 7.0, dissolved oxygen 30%) [6]. Similarly, l-arabinose, d-arabinose, and l-lyxose can be engineered to produce EG using similar pathways (Fig. 2b) [6]. Interestingly, the engineered strain can simultaneously utilize d-xylose and l-arabinose, two most abundant pentoses in lignocellulose, giving the promise to utilize raw materials such as lignocellulose to directly produce EG.

It should be noticed that all the three mentioned pentose utilization pathways are based on aldolases to cleave 5-carbon sugar or sugar phosphate into 2-carbon (glycolaldehyde) and 3-carbon (pyruvate or dihydroxyacetone phosphate) intermediates. Only glycolaldehyde can be utilized for EG production, while 3-carbon intermediate cannot be transferred into EG. Thus, the maximum yield of EG is only 1.0 mol/mol pentose (or 0.4 C-mol/C-mol) by these pathways, which is not economically feasible for EG industrialization. Moreover, glucose, the most abundant sugar, cannot be converted into EG. Thus, we have proposed two alternative routes for EG production based on the derivation of serine synthesis pathway (Fig. 2a) [40]. In the first route (route ① of Fig. 2a), serine is deaminated to hydroxypyruvate by transaminase or amino acid dehydrogenase. Hydroxypyruvate is then decarboxylated to glycolaldehyde by α-keto acid decarboxylase, which can be further converted to EG by alcohol dehydrogenase. In another pathway (route ② of Fig. 2a), serine is first converted into ethanolamine by serine decarboxylase and then oxidized to glycolaldehyde by monoamine oxidase. The maximum yield of EG can reach 0.69 g/g glucose (0.67 C-mol/C-mol glucose), which is much higher than that of ethanol (0.51 g/g glucose) [41]. These two metabolic pathways can be utilized to convert different carbon sources into EG. For example, the 3-carbon intermediate (dihydroxyacetone phosphate or pyruvate) generated in the previously mentioned pentose utilization pathways can be further converted to EG based on these routes, which significantly increases its maximum yield to 0.8 C-mol/C-mol pentose. Thus, it gives the promise to directly produce EG from renewable bioresources like lignocellulose. However, construction of a highly efficient strain based on the proposed pathways is challenging, due to (1) the lack of known enzymes to efficiently catalyze the designed reactions (serine decarboxylation and deamination, hydroxypyruvate decarboxylation, and ethanolamine oxidation) and (2) highly active serine degradation pathways in cellular metabolism. Different enzyme candidates of the proposed pathways have been screened and it was found that the combination of alanine-glyoxylate aminotransferase from *Arabidopsis thaliana* (AtAGT), benzoylformate decarboxylase from *Pseudomonas putida* (PpMdlC), and YqhD from *E. coli* was the most efficient for hydroxypyruvate-dependent EG synthetic route [40]. The combination of serine decarboxylase from *Arabidopsis thaliana* (AtSdc), amine oxidase from *Arthrobacter* sp. (AsAO), and YqhD from *E. coli* was the most efficient for ethanolamine-dependent EG synthetic route [40]. To enhance the availability of serine for EG synthesis, serine synthesis pathway was enhanced via the overexpression of the mutated *serA* gene encoding feedback-insensitive 3-phosphoglycerate dehydrogenase, *serB*, and *serC* genes encoding phosphoserine phosphatase and phosphoserine aminotransferase [42]. The serine degradation pathways were blocked via the deletion of *sdaA* gene encoding serine dehydratase, and *pabABC* genes encoding aminodeoxychorismate synthase and aminodeoxychorismate lyase. The engineered *Corynebacterium glutamicum* can produce 3.5 g/L EG with a yield of 0.09 g/g glucose [40]. Similar strategies have also been applied to *E. coli*, resulting in a strain that can produce 4.1 g/L EG with a yield of 0.14 g/g glucose [43]. The titer and yield of EG in these studies are still far from the requirement of industrialization. The main reason for the low titer and yield is probably the low activities of the screened enzymes. Thus, protein engineering to enhance the activities of the pathway enzymes and fine-tuning of gene expression for pathway balancing are important for further optimization of EG production.

Recently, the potential pathways for the synthesis of EG from syngas have been explored based on the BNICE pathway prediction system [7]. It was found that there exist many potential pathways and the maximum yield of EG can reach 0.74 g/g H_2_ + CO_2_ or 0.44 g/g CO. For these pathways, syngas is first converted into acetyl-CoA via the Wood–Ljungdahl pathway of acetogenic bacteria, and acetyl-CoA can be further transferred into EG via gluconeogenesis and other pathways. However, all of these pathways have not been experimentally verified.

Due to the low price of EG, substantial efforts should be made to significantly reduce the production cost of bio-based EG. To be competitive with chemical processes, the titer, yield, and productivity of bio-EG should be higher than 100 g/L, 0.5 g/g, and 3.0 g/L/h, respectively. Thus, there is a long way towards the commercialization of bio-EG.

## Metabolic engineering for the production of C3 diols

There are two isomers of C3 diols, namely 1,3-PDO and 1,2-PDO. Both diols have wide industrial application and large market potentials. 1,3-PDO can be used as a solvent, an antifreeze, and a monomer for the synthesis of polyethers, polyurethanes, and polyesters [1, 44]. Especially, 1,3-PDO can be used to produce PTT, a new type of polyester which has better properties than PET, e.g., better tension and elasticity. Biological production of 1,3-PDO has already been commercialized and almost completely substituted the chemical processes. 1,2-PDO is a commodity chemical with a global demand of around 1.5 million tons/year. It can be used as a solvent, an antifreeze, a preservative, an ingredient, and a monomer for the production of unsaturated polyester resins [45]. Currently, 1,2-PDO is produced from propylene oxide derived from petroleum industry via chemical processes. There is increasing interest to directly produce 1,2-PDO from renewable bioresources by biological processes.

### 1,3-Propanediol

**Fig. 3:**
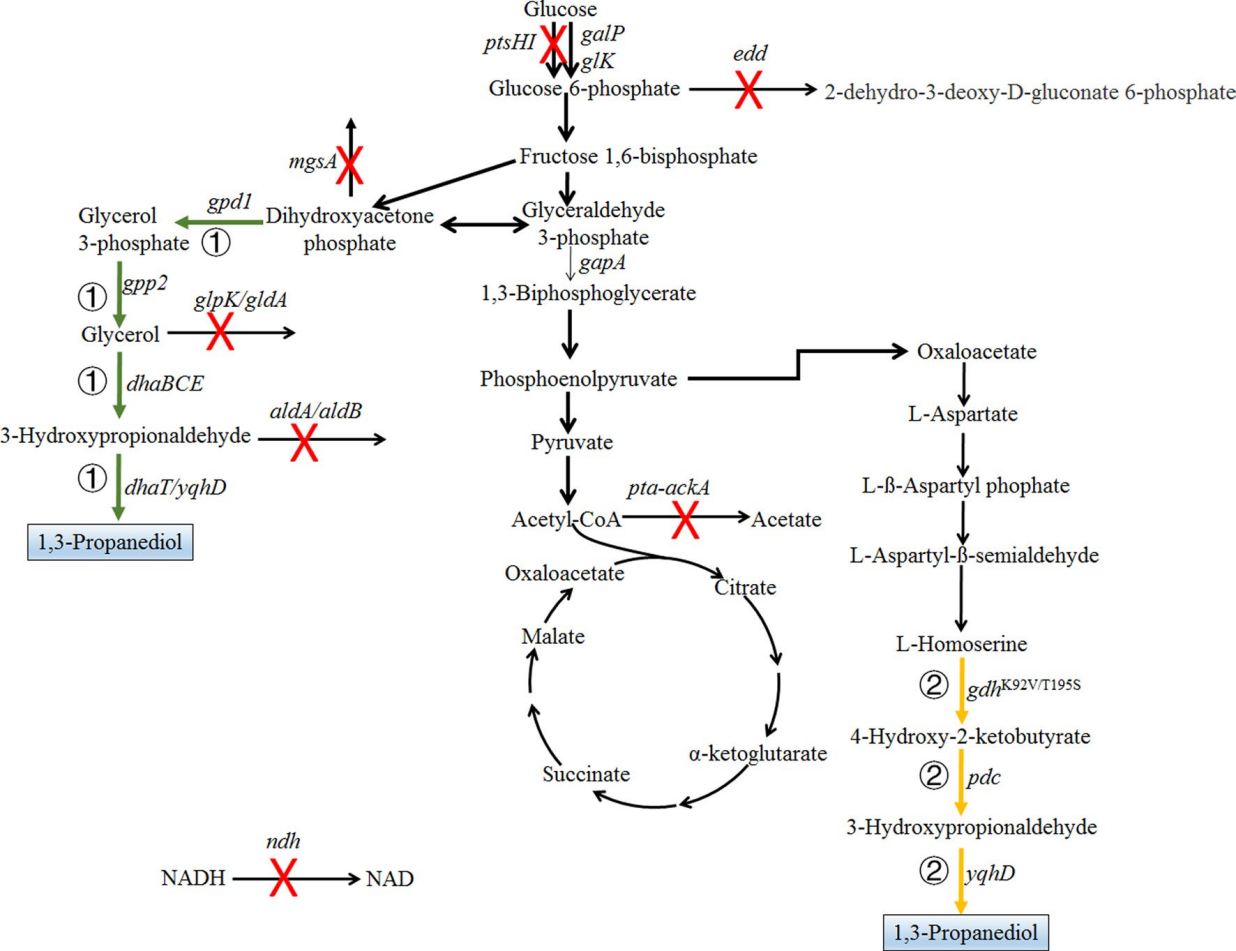
Metabolic pathways and metabolic engineering strategies for the production of 1,3-propanediol (1,3-PDO). 1,3-PDO can be produced from glucose by glycerol-dependent pathway (route ①, illustrated by green colors) or by a non-natural homoserine-derived pathway (route ②, illustrated by yellow colors). To generate a 1,3-PDO hyperproducer via glycerol-dependent pathways, different byproduct synthetic pathways should be blocked as illustrated by red cross marks. *ackA* acetate kinase, *aldA/aldB* aldehyde dehydrogenases, *edd* 6-phosphogluconate dehydratase, *galP* galactose-proton symporter, *glk* glucose kinase, *gdh* ^K92V/T195S^ glutamate dehydrogenase with point mutation of K92V/T195S, *gpd1* glycerol 3-phosphate dehydrogenase, *gpp2* glycerol 3-phosphate phosphatase, *gldA* glycerol dehydrogenase, *glpK* glycerol kinase, *mgsA* methylglyoxal synthase, *ndh* NADH dehydrogenase II, *pdc* pyruvate decarboxylase, *pta* phosphate acetyltransferase, *ptsHI* components of phosphoenolpyruvate-dependent phosphotransferase system, *yqhD/dhaT* alcohol dehydrogenase

1,3-PDO is a natural product during the anaerobic degradation of glycerol by several microbes, such as *Klebsiella pneumoniae*, *Clostridium butyricum*, *Enterobacter agglomerans*, *Citrobacter freundii*, and *Lactobacillus brevis* [46–49]. In this process, glycerol is dehydrated to 3-hydroxypropionaldehyde (3-HPA) by glycerol dehydratase, and the latter is further reduced to 1,3-PDO by alcohol dehydrogenase with the consumption of 1 mol NADH (Fig. 3) [47]. The reducing equivalent is regenerated via glycerol oxidation pathway, resulting in the formation of different byproducts (acetate, lactate, ethanol, 2,3-butanediol, succinate, etc.). Different strategies have been attempted to modify these natural producers in order to increase the production of 1,3-PDO and to reduce the accumulation of byproducts [16]. Several comprehensive reviews for 1,3-PDO production from glycerol have been published elsewhere and will not be further expanded here [1, 8, 16]. Most previous metabolic engineering efforts focused on blocking only one or several byproduct synthesis pathways (such as lactate and ethanol) and were not efficient to increase the production of 1,3-PDO due to the high flexibility of glycerol oxidation pathways [50–52]. Since it is not possible to block all byproduct synthesis pathways due to the necessity of glycerol oxidation for NADH regeneration and cell growth, a strategy for the co-production of 1,3-PDO and d-lactate has been proposed by Xin et al. [53]. The conversion of glycerol to d-lactate is an oxidative process which generates 1 mol NADH per mol d-lactate, and thus cofactor can be well recycled during the co-production process. With the block of metabolic pathways to acetate, ethanol, 2,3-butanediol, and succinate (Δ*ackA*-*pta*Δ*poxB*Δ*adhE*Δ*budAB*Δ*frdA*), the engineered *K. oxytoca* can produce 76 g/L 1,3-PDO and 112 g/L d-lactate with a conversion yield of 0.95 mol/mol glycerol in fed-batch fermentation [53]. 1,3-PDO and d-lactate are both valuable products which can be easily separated in the downstream process, and therefore the co-production strategy potentially revitalizes the whole biorefinery process with the higher atomic economy and the lower production cost. We also proposed a similar approach for the co-production of 1,3-PDO with glutamate using glycerol and glucose as co-substrates [54]. Glutamate is synthesized from glucose with the generation of extra NADH for 1,3-PDO formation from glycerol. With the introduction of 1,3-PDO synthesis pathway into an industrial glutamate-producing *C. glutamicum*, the engineered strain can efficiently convert glycerol and glucose to their targeted products 1,3-PDO and glutamate. Due to the lack of glycerol oxidation pathway in *C. glutamicum*, 1,3-PDO was produced with extremely high yield (~ 1.0 mol/mol glycerol). At the same time, the yield of glutamate to glucose was also increased owing to efficient cofactor regeneration. Since 1,3-PDO and glutamate can also be easily separated, the proposed process may be integrated into current glutamate production system to increase the whole process economy. Co-production of 1,3-PDO with 3-hydroxypropionic acid (3-HP) is also a promising approach for cofactor recycling to increase the process economy. With the introduction of an aldehyde dehydrogenase and block of lactate, ethanol, and succinate synthesis pathways, an engineered *K. pneumoniae* can co-produce 1,3-PDO and 3-HP with the yield of 0.82 mol/mol glycerol with additional B_12_ supplementation [55].

There is no natural microorganism which can directly produce 1,3-PDO from sugars. Since there are natural pathways for the synthesis of both glycerol and 1,3-PDO, these two modules can be combined in one recombinant cell. The most successful work was done by Dupont who reconstructed a recombinant *E. coli* that can utilize glucose to produce 1,3-PDO with a titer of 135 g/L and a productivity of 3.5 g/L/h in aerobic fermentation process [17]. The whole synthetic metabolic pathway is simple: the glycerol synthesis module consisting of glycerol 3-phosphate dehydrogenase (GPD1) and glycerol 3-phosphate phosphatase (GPP2), and the 1,3-PDO synthesis module consisting of glycerol dehydratase and an alcohol dehydrogenase (route ① of Fig. 3). The theoretical yield of PDO from sugar is 1.5 mol/mol glucose without considering cell growth and energy maintenance. To achieve high yield and productivity satisfying the industrial requirement, protein engineering and systems metabolic engineering should be intensively integrated during the strain and process development. At protein and pathway levels, heterologous proteins should be modified for concerted action to increase pathway efficiency. For glycerol synthesis module, it was found that the formation of a fusion protein of GPD1 and GPP2 in recombinant *E. coli* during strain evolution could significantly increase the production of glycerol due to partial glycerol 3-phosphate channeling between the active sites of the two proteins [56]. For 1,3-PDO synthesis module, both glycerol dehydratase and alcohol dehydrogenase should be engineered. Firstly, the B_12_-dependent glycerol dehydratase (encoded by *dhaBCE* genes of *K. pneumoniae*) consisting of three subunits is highly sensitive to oxygen and is inhibited by both glycerol and 1,3-PDO [57, 58]. Protein engineering of glycerol dehydratase by error-prone PCR and high-throughput screening has enabled the identification of several variants showing high resistance to oxygen, glycerol, and 1,3-PDO [59]. Interestingly, it was found that the formation of a fusion protein between subunits α and β of glycerol dehydratase is the main reason for the improved properties, indicating that metabolic channeling may also play an important role in the recombinant glycerol dehydratase. Furthermore, overexpression of *butR* gene encoding cob(I)alamin adenosyltransferase to enhance the transformation of B_12_ to coenzyme B_12_, an essential cofactor of glycerol dehydratase, also increased the production of 1,3-PDO [60]. For alcohol dehydrogenase, it was found that the NADH-dependent 1,3-PDO dehydrogenase (PDOR) from *K. pneumoniae* was not effective in *E. coli* under the aerobic condition as compared to NADPH-dependent alcohol dehydrogenase YqhD from *E. coli*, probably due to the high reversibility of reaction catalyzed by PDOR [17, 61]. Thus, a recombinant *E. coli* strain without PDOR showed much higher production of 1,3-PDO in fed-batch fermentation as compared to the one with PDOR overexpression (112 g/L vs 68 g/L) [61].

Besides terminal pathway engineering, efficient engineering of central metabolism to increase the availability of precursor and reducing equivalent is also highly important for 1,3-PDO production from sugar. There are several pathways in *E. coli* which divert the carbon flux from 1,3-PDO synthesis. First, the pathway intermediate glycerol can be channeled into central glycolysis via glycerol dehydrogenase (*gldA*) and glycerol kinase (*glpK*) [62, 63]. A recombinant *E. coli* strain with the deletion of *gldA* and *glpK* genes and overexpression of glycerol and 1,3-PDO synthesis pathway can produce 112 g/L 1,3-PDO with a yield of 0.26 g/g glucose [61]. Since the fermentation of 1,3-PDO by the recombinant *E. coli* was carried out under aerobic condition, most of generated NADH was consumed via oxidative phosphorylation. Thus, deletion of *ndh* gene encoding non-proton pumping type II NADH dehydrogenase of the mentioned strain significantly increased the yield of 1,3-PDO to 0.34 g/g glucose [61]. Deletion of *aldA* and *aldB* genes encoding aldehyde dehydrogenases to prevent the oxidation of 3-HPA, and *mgsA* gene encoding methylglyoxal synthase to prevent the conversion of dihydroxyacetone phosphate to methylglyoxal, could also slightly increase the yield of 1,3-PDO [61]. Second, since *E. coli* utilizes phosphoenolpyruvate (PEP)-dependent phosphotransferase system (PTS) as the main route for glucose uptake, the production of 1,3-PDO is coupled with the requirement of PEP synthesis, which results in inevitably high flux allocation toward the downstream pathway of glycolysis and reduced yield of 1,3-PDO. Thus, the substitution of PEP-dependent glucose transport system by the non-PEP-dependent system is an important strategy to increase the yield of 1,3-PDO and other metabolites [63]. Deletion of *ptsHI* gene and overexpression of *galP* gene encoding galactose-proton symporter and *glk* gene encoding glucose kinase further increased the yield of 1,3-PDO to 0.42 g/g (from 0.34 g/g) [61]. Third, reduction of flux from glyceraldehyde 3-phosphate to the downstream pathway of glycolysis is necessary to further increase the yield of 1,3-PDO. Since glycolysis pathway is necessary for cell growth, down-regulation of glyceraldehyde 3-phosphate dehydrogenase encoded by *gapA* gene by promoter substitution can be applied. The yield of 1,3-PDO can be increased to 0.46 g/g (from 0.34 g/g) via a choice of suitable promoter for *gapA* gene [61]. Other potential strategies to further modify this strain include the deletion of *edd* gene encoding 6-phosphogluconate dehydratase to block the Entner–Doudoroff (ED) pathway, deletion of *pta*-*ackA* to reduce acetate accumulation, and deletion of *arcA* gene encoding aerobic respiration protein to reduce the regulation of glycolysis genes. The final engineered strain contained 15 different modifications of *E. coli* genes (deletion of *glpK*, *gldA*, *ndh*, *ptsHI*, *edd*, *arcA*, *ptaA*-*ackA*, *aldA*, *aldB*, and substitution of the promoters of *galP*, *glk*, *ppc*, *yqhD*, *btuR*, and *gapA*), which can produce 1,3-PDO with a yield of 0.49 g/g glucose (1.2 mol/mol) corresponding to 77% of the maximum yield [61, 64].

Although glycerol-dependent pathway has been successfully implemented for 1,3-PDO production from glucose, this process requires the addition of expensive vitamin B_12_ during the fermentation, which increases the whole production cost [65]. We have proposed a glycerol-independent non-natural pathway which does not need the addition of vitamin B_12_ (route ② of Fig. 3) [66]. This pathway utilizes homoserine as a precursor: first, homoserine is transferred to 4-hydroxy-2-ketobutyrate by amino acid dehydrogenase or transaminase; 4-hydroxy-2-ketobutyrate is further transferred to 3-HPA by α-keto acid decarboxylase; and 3-HPA can be reduced to 1,3-PDO by alcohol dehydrogenase. The maximum yield of this pathway is the same as that of the glycerol-dependent pathway (1.5 mol/mol glucose). The advantage of this pathway is that it does not contain any enzyme that requires a complicated cofactor, and thus simple medium can be applied. We have proved the feasibility of this pathway in *E. coli* with the introduction of modified glutamate dehydrogenase (K92V/T195S), pyruvate decarboxylase from *Zymomonas mobilis*, and YqhD from *E. coli* [66]. Since homoserine is an important intermediate in threonine synthesis pathway, the proposed non-natural pathway can be integrated into high threonine producer with the deletion of homoserine kinase to improve the production of 1,3-PDO [67–69].

Although biological production of 1,3-PDO has been commercialized, the production cost is still relatively high which limits its wide application. Due to the sharp reduction of crude glycerol price, development of efficient glycerol-based 1,3-PDO production system is becoming promising. Systems metabolic engineering should be applied to further reduce the byproduct formation to simplify the downstream process. Moreover, introduction or modification of B_12_ synthesis pathway to get rid of B_12_ supplementation is also an important issue for the reduction of production cost.

### 1,2-Propanediol

**Fig. 4:**
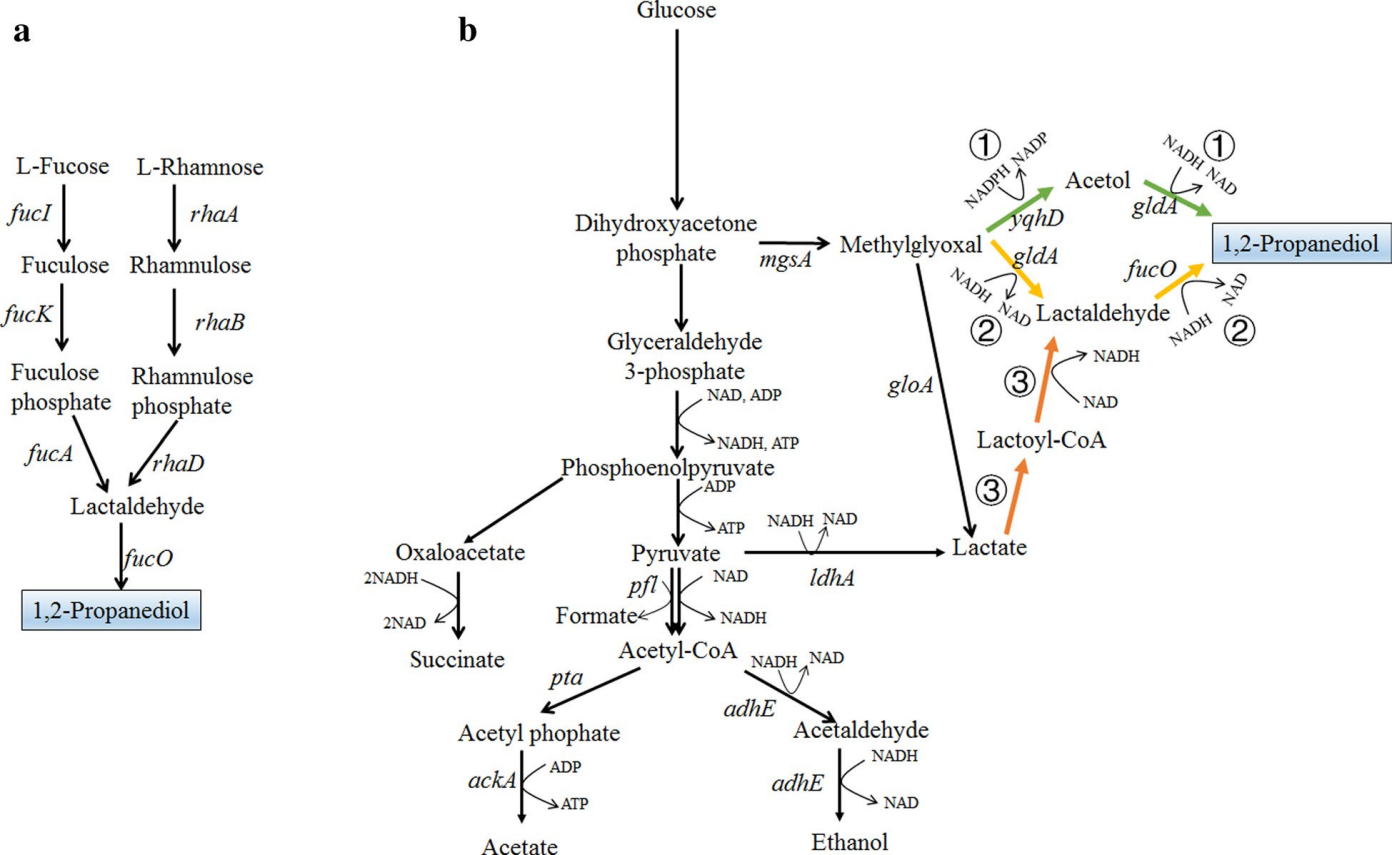
Metabolic pathways for the production of 1,2-propanediol (1,2-PDO). **a** Metabolic pathways for the production of 1,2-PDO from l-fucose and l-rhamnose; **b** metabolic pathways for the production of 1,2-PDO from glucose. Three pathways have been proposed to convert glucose into 1,2-PDO via either methylglyoxal (route ① illustrated by green colors and route ② illustrated by yellow colors) or lactate (route ③ illustrated by orange colors). *ackA* acetate kinase, *adhE* acetaldehyde/alcohol dehydrogenase, *fucA* d-fuculose 1-phosphate aldolase, *fucI* l-fucose isomerase, *fucK* fuculokinase, *fucO* NADH-dependent alcohol dehydrogenase, *gloA* glyoxalase I, *ldhA* lactate dehydrogenase, *mgsA* methylglyoxal synthase, *pfl* pyruvate formate lyase, *pta* phosphate acetyltransferase, *rhaA* l-rhamnose isomerase, *rhaB* l-rhamnulokinase, *rhaD* d-rhamnulose 1-phosphate aldolase

1,2-PDO is another important C3 diol with high industrial demand. It contains an asymmetrical carbon atom and has two stereoisomers: *R*-1,2-PDO and *S*-1,2-PDO. The commercial product from chemical processes is a racemic mixture. The pure stereoisomers can be used as chiral synthons in organic synthesis [9]. Several microorganisms such as *Clostridium thermosaccharolyticum* can naturally utilize l-rhamnose and l-fucose to synthesize 1,2-PDO [70]. In this process, l-rhamnose or l-fucose is transferred to lactaldehyde by an isomerase, a kinase, and an aldolase (Fig. 4a). Lactaldehyde is finally reduced to 1,2-PDO by an alcohol dehydrogenase. The direct conversion of these expensive carbon sources to bulk chemicals like 1,2-PDO, however, is not economically feasible. Thus, a more applicable route based on the reduction of methylglyoxal has been proposed and widely investigated in different chassis [9, 71, 72]. In this metabolic route, dihydroxyacetone phosphate is first converted to methylglyoxal by methylglyoxal synthase (*mgsA*). Methylglyoxal can be transferred to 1,2-PDO via two alternative pathways, with either acetol or lactaldehyde as an intermediate through the concerted action of an alcohol dehydrogenase (*fucO* or *yqhD*) and a glycerol dehydrogenase (*gldA*) (route ① and ② of Fig. 4b). This metabolic route can transfer different substrates, including sugars, glycerol, or CO_2_, into 1,2-PDO [9, 71, 72]. Three key challenges should be solved toward the construction of a 1,2-PDO hyperproducer: (1) cofactor regeneration; (2) accumulation of byproducts; and (3) accumulation of toxic intermediates. Like 1,3-PDO, the production of 1,2-PDO from glucose or glycerol is a reductive process which consumes NAD(P)H. For the anaerobic or microaerobic production of 1,2-PDO, coupling the production of acetate with 1,2-PDO would give the highest yield of 1,2-PDO [9, 10, 73]. To increase the availability of NADH and to reduce byproduct formation, it is necessary to block the synthesis of lactate, ethanol, and succinate. Overexpression of formate dehydrogenase for the oxidation of formate is also an effective approach for NADH generation [10]. Together with the overexpression of *fdhI* gene encoding formate dehydrogenase from *Candida boidinii*, a recombinant *E. coli* strain with the deletion of *zwf* (glucose 6-phosphate dehydrogenase), *ldhA, gloA* (glyoxalase I), and *adhE* genes can produce 0.59 g/L 1,2-PDO with a yield of 0.34 g/g glucose [10]. The low titer of 1,2-PDO may be due to metabolic imbalance and slow cell growth. Further laboratory adaptation of the engineered strain improved the titer and yield to 5.13 g/L and 0.48 g/g glucose after 120 h [10]. Compared to anaerobic cultivation, the aerobic fermentation can significantly increase cell growth and productivity. *C. glutamicum* has been engineered to produce 1,2-PDO from glucose in aerobic condition via the introduction of *mgsA, gldA, and yqhD* from *E. coli* [74]. *YqhD* gene was found to be more efficient for 1,2-PDO production than *fucO* under aerobic condition, probably due to the more abundant NADPH in *C. glutamicum* under aerobic condition. With the deletion of *ldhA* and *hdpA* genes to block the synthesis of lactate and glycerol, the strain can produce 4.8 g/L 1,2-PDO with a yield of 0.15 g/g glucose [74]. As discussed in the previous section, a significant proportion of NADH is oxidized via oxidative phosphorylation under aerobic condition, and thus the down-regulation of oxidative phosphorylation via the deletion of *ndh* or other related genes could be applied to increase the yield of 1,2-PDO. Similarly, the strategies applied for 1,3-PDO production, such as substitution of PEP-dependent glucose transport system by non-PEP-dependent system and down-regulation of glyceraldehyde 3-phosphate, could also be applied to engineer the 1,2-PDO-producing strain. Production of 1,2-PDO from CO_2_ has been engineered in *Synechococcus elongatus* which can accumulate ~ 150 mg/L of 1,2-PDO in aerobic condition [72]. The key improvement was obtained by the utilization of NADPH-dependent secondary alcohol dehydrogenase and NADPH-dependent YqhD to substitute the NADH-dependent glycerol dehydrogenase and FucO, also due to the more abundance of NADPH in *S. elongatus*.

The methylglyoxal-based 1,2-PDO synthesis pathways contain several toxic intermediates, especially methylglyoxal and lactaldehyde. Thus, engineering metabolic balance of the pathway to minimize the accumulation of toxic intermediates is highly important for increasing the productivity of 1,2-PDO. Several new strategies based on DNA scaffolding and bacterial microcompartment (BMC) have been tried. For example, Conrado et al. developed a process to fuse zinc-finger domains to the enzymes of 1,2-PDO synthesis pathway [75]. The zinc-finger domains can specifically bind DNA scaffold and thus permit spatial and temporal organization of enzymes to reduce intermediate diffusion [75]. Under optimized enzyme/scaffold ratio, the production of 1,2-PDO was improved by ~ 3.5 times compared to the control strain with free enzymes. Microcompartment is another smart system that has been used by microbes to address the problem of unstable or reactive intermediates by encapsulating pathway enzymes within a protein shell [76]. Pdu BMC is a natural microcompartment that functions in the 1,2-PDO degradation process [77]. Lee et al. tried to construct an artificial BMC for 1,2-PDO synthesis by adding N-terminal targeting sequences derived from Pdu BMC to the enzymes of 1,2-PDO synthesis pathway in *E. coli* [78]. The strain containing the fused enzymes exhibited a 245% increase of 1,2-PDO production in comparison to the strain with free enzymes.

A methylglyoxal-independent 1,2-PDO synthesis pathway has recently been proposed to avoid the generation of toxic intermediates [79]. This pathway uses lactate as a metabolic precursor, which is transferred to lactoyl-CoA by a lactoyl-CoA transferase and further reduced to lactaldehyde by CoA-acylating aldehyde dehydrogenase and 1,2-PDO by alcohol dehydrogenase (route ③ of Fig. 4b). Depending on the stereochemistry of lactate, different 1,2-PDO stereoisomers can be produced. With the deletion of lactate utilization genes *lldD* and *dld*, a recombinant *E. coli* can produce 1.5 g/L *R-*1,2-PDO from d-lactate and 1.7 g/L *S-*1,2-PDO from l-lactate with high enantiomeric purity (> 98% ee), with the combination of CoA transferase gene *pct* from *Megasphaera elsdenii*, aldehyde dehydrogenase gene *pduP* from *Salmonella enterica*, and alcohol dehydrogenase gene *yahK* from *E. coli* [79]. Similarly, Zhu et al. constructed a complete lactate-based pathway for direct conversion of glucose to *S*-1,2-PDO with a titer of 1.04 g/L with further modifications, including overexpression of l-lactate dehydrogenase from *Bacillus coagulans*, blocking the d-lactate synthesis (Δ*ldhA*), lactate utilization (Δ*dld*), acetate (Δ*ackA*-*pta*Δ*poxB*) and ethanol (Δ*adhE*) synthesis pathways [80].

As a bulk chemical, the price of 1,2-PDO is much lower than that of 1,3-PDO. However, the titers, yields, and productivities of 1,2-PDO by most of the reported work are much lower than those of 1,3-PDO. Thus, substantial efforts should be made to increase the strain performance toward a feasible 1,2-PDO bioproduction process.

## Metabolic engineering for the production of C4 diols

There are four isomers of C4 diols, including 1,4-BDO, 2,3-BDO, 1,3-BDO, and 1,2-BDO. All of these isomers show wide industrial application. 1,4-BDO is one of the most important diols with an annual production of more than 1 million tons [81]. It is widely used in industry as a solvent and a monomer for the synthesis of plastics, polyesters, and spandex fibers. 2,3-BDO is considered as a potential platform chemical which can be used as biofuel and a building block for the synthesis of 2-butanone [82], 2-butanol [83], and 1,3-butadiene [84]. 1,3-BDO is widely used as a solvent in food and cosmetic industries [85]. 1,2-BDO is used to produce polyester resins and plasticizers. Most of the C4 diols (1,4-BDO, 1,3-BDO, and 1,2-BDO) are non-natural metabolites which lack natural synthetic pathways. In recent years, different new pathways have been proposed for the synthesis of 1,4-BDO and 1,3-BDO [81, 85, 86]. Especially, biological production of 1,4-BDO from sugars has been commercialized by Genomatica [23]. So far, no metabolic pathways for 1,2-BDO synthesis have been reported. In this section, we will review the recent advances in metabolic engineering to produce 1,4-BDO, 2,3-BDO, and 1,3-BDO.

### 1,4-Butanediol

**Fig. 5:**
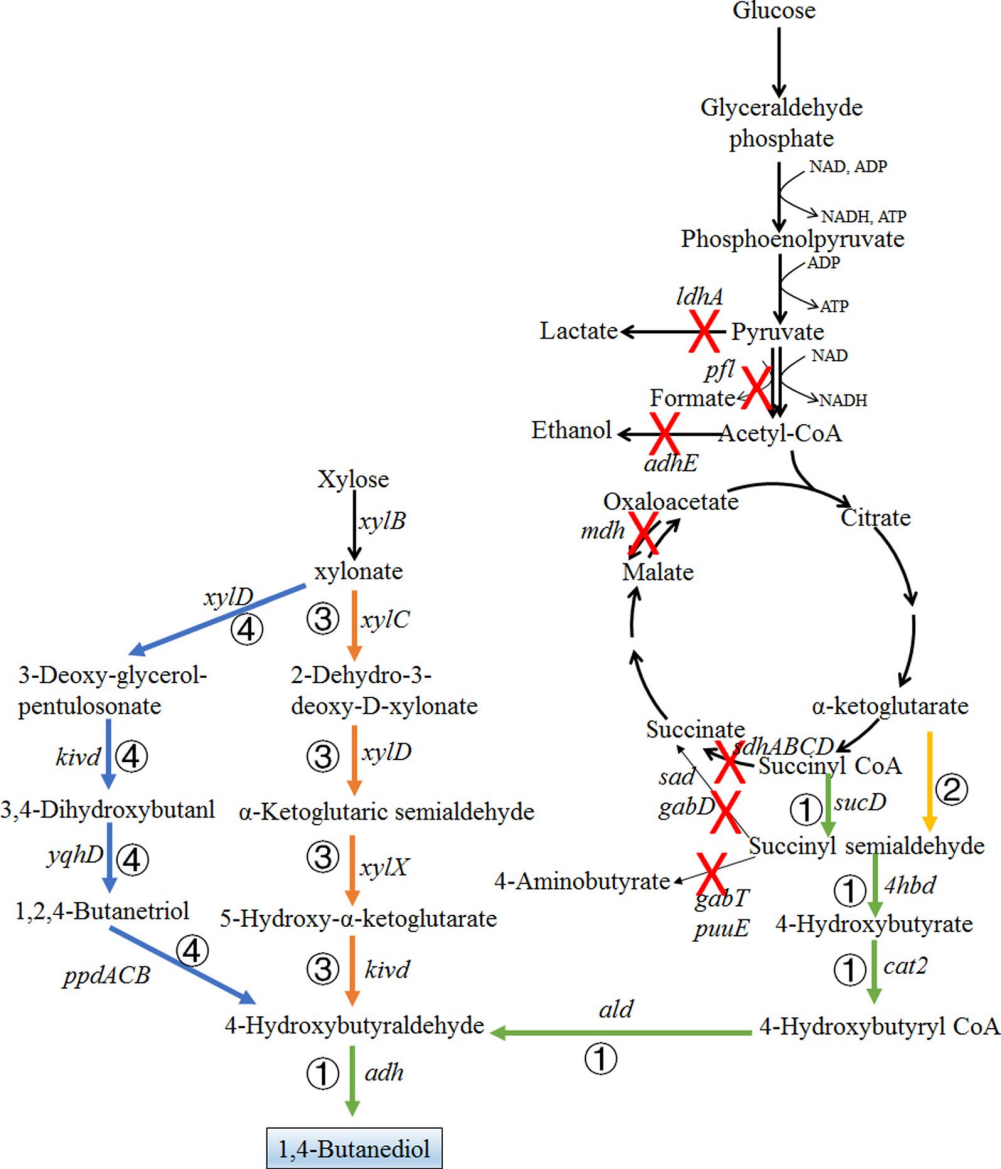
Metabolic pathways and metabolic engineering strategies for the production of 1,4-butanediol (1,4-BDO). Two metabolic pathways have been proposed to convert glucose into 1,4-BDO via succinyl semialdehyde. Route ① (illustrated by green colors) starts from succinyl-CoA, which is converted into succinyl semialdehyde by succinyl-CoA dehydrogenase. Route ② (illustrated by yellow color) starts from α-ketoglutarate, which is converted into succinyl semialdehyde by keto acid decarboxylase. Succinyl semialdehyde can be converted into 1,4-BDO in four steps. Xylose can also be converted into 1,4-BDO by two proposed pathways (route ③ illustrated by orange colors and route ④ illustrated by blue colors). To generate a 1,4-PDO hyperproducer from glucose via route ① and route ②, different byproduct synthetic pathways should be blocked as illustrated by red cross marks. *adh* alcohol dehydrogenase, *ald* 4-hydroxybutyrate-CoA reductase, *adhE* acetaldehyde/alcohol dehydrogenase, *cat2* 4-hydroxybutyrate-CoA transferase, *gabD* succinate semialdehyde dehydrogenase, *gabT/puuE* aminotransferase, *4hbd* 4-hydroxybutyrate dehydrogenase, *kivd* α-ketoisovalerate decarboxylase, *ldhA* lactate dehydrogenase, *mdh* malate dehydrogenase, *pfl* pyruvate formate lyase, *ppdACB* glycerol dehydratase, *sad* succinate semialdehyde dehydrogenase, *sdhABCD* succinate dehydrogenase, *sucD* succinyl-CoA dehydrogenase, *xylB* d-xylose dehydrogenase, *xylC* d-xylonate dehydratase, *xylD* 2-dehydro-3-deoxy-d-xylonate dehydratase, *xylX* α-ketoglutaric semialdehyde dehydrogenase, *yqhD* NAD(P)H-dependent alcohol dehydrogenases

Since there is no natural pathway for the direct synthesis of 1,4-BDO, metabolic pathway prediction algorithms should be used to predict the potential synthesis routes. Genomatica has used their in-house developed software called as SimPheny Biopathway Predictor to identify more than 1000 potential pathways of four to six steps for the synthesis of 1,4-BDO from common central metabolites [23]. The basic principle of SimPheny is similar to BNICE, which is based on generalized enzyme reaction rules to find potential connection routes [23, 27]. Two metabolic routes were selected: one starts from α-ketoglutarate (route ① of Fig. 5), and the other starts from succinyl-CoA (route ② of Fig. 5). Both α-ketoglutarate and succinyl-CoA can be converted to succinyl semialdehyde by α-keto acid decarboxylase or succinyl-CoA dehydrogenase (*sucD*). Succinyl semialdehyde is further converted to 4-hydroxybutyrate by an aldehyde reductase. 4-hydroxybutyrate can be transferred to 1,4-BDO by a CoA transferase, an aldehyde dehydrogenase, and an alcohol dehydrogenase. It was found that succinyl-CoA-based pathway is more efficient than α-ketoglutarate-based pathway due to the low activity of α-ketoglutarate decarboxylase [23]. To engineer a highly efficient 1,4-PDO producer, systems approaches need to be used for process diagnosis and to solve three most important challenges: (1) screening and engineering of efficient enzymes to build the non-natural 1,4-BDO pathway; (2) cofactor regeneration; and (3) reduction of byproducts [22, 87]. Several reactions within this metabolic pathway lack natural specific enzymes, such as 4-hydroxybutyrate dehydrogenase (*4hbd*), 4-hydroxybutyrate-CoA transferase (*cat2*), 4-hydroxybutyrate-CoA reductase (*ald*), and 1,4-BDO dehydrogenase (*adh*). Thus, enzyme candidates for these steps were first screened via bioinformatic analysis of known enzymes that catalyze analogous transformations. It was found that the combination of *4hbd* and *cat2* from *Porphyromonas gingivalis*, *ald* from *C. beijerinckii*, and an inherent *adh* from *E. coli* gave the highest production of 1,4-BDO by *E. coli* [23]. It should be noticed that acetyl-CoA can also be transferred to ethanol by *ald* and *adh* due to their broad substrate specificity. Thus, protein engineering was further utilized to engineer *ald* to improve its activity and specificity toward 4-hydroxybutyrate-CoA, and to reduce the production of ethanol [22, 87, 88]. Moreover, protein engineering was also applied to reduce the inhibition of *cat2* by a high concentration of 1,4-BDO, which consequently reduced the accumulation of 4-hydroxybutyrate and increased the production rate of 1,4-BDO at the later stage of fermentation [22, 87].

Besides a functional 1,4-BDO pathway in *E. coli*, the central metabolism of *E. coli* should also be intensively modified. The production of 1,4-BDO from glucose is a highly reductive process which consumes 4 mol NAD(P)H per mol 1,4-BDO. Thus, reducing equivalent should be efficiently generated. With the deletion of alcohol dehydrogenase (*adhE*), pyruvate formate lyase (*pfl*), lactate dehydrogenase (*ldh*), and malate dehydrogenase (*mdh*) genes to block the synthesis of ethanol, formate, lactate, and succinate, the metabolic flux and NADH should be channeled to the oxidative branch of TCA cycle for 1,4-BDO synthesis. However, high accumulation of pyruvate and acetate was observed. The potential reason is that pyruvate dehydrogenase and citrate synthase are inhibited by high concentration of NADH. Thus, replacement of the inherent pyruvate dehydrogenase E3 subunit gene *lpdA* by a mutant *lpdA* (D354K) from *K*. *pneumoniae* and introduction of a point mutation of R163L into citrate synthase to improve their activities and resistance to NADH improved cell growth and 1,4-BDO production [22, 87]. Reduction of NADH consumption in oxidative phosphorylation via the deletion of *ndh* gene as mentioned before was also helpful to improve the yield of 1,4-BDO. Further improvement of 1,4-BDO production was achieved with the combination of omics tools, which identified the following targets: (1) deletion of *sad* and *gabD* genes encoding succinate semialdehyde dehydrogenases to block the conversion of succinyl semialdehyde to succinate; (2) deletion of *gabT* and *puuE* genes encoding aminotransferase enzymes to block the conversion of succinyl semialdehyde to 4-aminobutyrate; and (3) deletion of *zwf* gene encoding glucose 6-phosphate dehydrogenase and *sdhABCD* gene encoding succinate dehydrogenase to block oxidative PPP and TCA cycle to reduce the generation of CO_2_ [22, 87]. A more detailed description of the strategies employed for the development of industrial 1,4-BDO producer can refer to a recent review by Burgard et al. [22]. The best engineered strain can produce 1,4-BDO with titer > 120 g/L, rate > 3.5 g/L/h, and yield > 0.4 g/g glucose (80% of theoretical).

Besides the mentioned novel pathway for the conversion of sugars to 1,4-BDO, a non-phosphorylated pathway for the conversion of xylose to 1,4-BDO has been proposed (route ③ of Fig. 5) [89, 90]. In this pathway, d-xylose is first oxidized to d-xylonate by d-xylose dehydrogenase (*xylB*); d-xylonate is then converted to α-ketoglutaric semialdehyde by two dehydratases, d-xylonate dehydratase (*xylC*) and 2-dehydro-3-deoxy-d-xylonate dehydratase (*xylD*); α-ketoglutaric semialdehyde is further reduced to 5-hydroxy-α-ketoglutarate by a dehydrogenase (*xylX*), where the latter can be converted to 1,4-BDO by a α-keto acid decarboxylase (*mdlC/kivd*) and an alcohol dehydrogenase (*adh*). The first four enzymes can be found from *Caulobacter crescentus* which are encoded by *xylBCDX* genes. Thus, only the last two enzymes should be screened. It was found that the combination of *kivd* from *Lactococcus lactis* and *yqhD* from *E. coli* was the most efficient [89]. It should be noticed that the enzymes encoded by *xylD*, *kivd*, and *yqhD* have promiscuous activities which can convert d-xylonate into 1,2,4-butanetriol via another route (route ④ of Fig. 5) [91]. Thus, the accumulation of 1,4-BDO, as well as 1,2,4-butanetriol, was observed during the fermentation. One approach to reduce the accumulation of 1,2,4-butanetriol is to alter the specificity of the mentioned enzymes. With the introduction of one point mutation in *kivd* (V461I), the accumulation of 1,2,4-butanetriol was reduced by 72.2%, while the production of 1,4-BDO was increased by 109.3% [89]. Alternatively, 1,2,4-butanetriol can be further converted to 4-hydroxybutyraldehyde by modified glycerol dehydratase [91]. The engineered strain with modified *kivd* and further deletion of *xylA*, *yjhH*, and *yagE* genes to block the consumption of d-xylose and d-xylonate can produce 12 g/L 1,4-BDO in fed-batch fermentation using xylose and glucose as the co-substrates [89]. Similar pathways can also be engineered for the conversion of l-arabinose and d-galacturonate to 1,4-BDO [89].

### 2,3-Butanediol

**Fig. 6:**
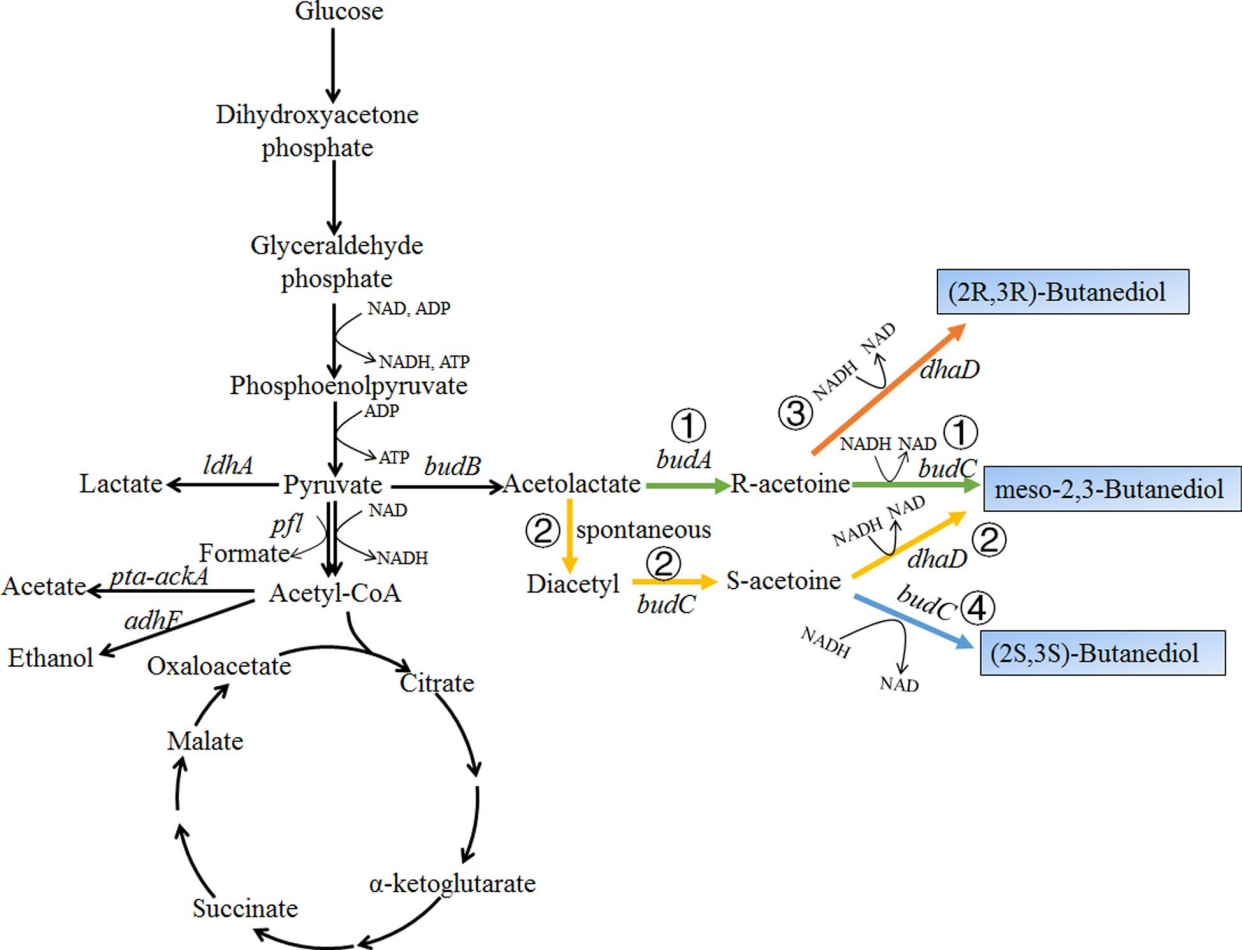
Metabolic pathways for the production of 2,3-butanediol (2,3-BDO). Different stereoisomers of 2,3-BDO can be produced from glucose by four metabolic pathways via acetolactate. Meso-2,3-BDO can be synthesized from acetolactate by acetolactate decarboxylase and *S-*specific 2,3-BDO dehydrogenase (route ①, illustrated by green colors) or from diacetyl by diacetyl reductase and *S-*specific 2,3-BDO dehydrogenase (route ②, illustrated by yellow colors). (2*R*,3*R*)-BDO can be synthesized from acetolactate by acetolactate decarboxylase and *R*-specific 2,3-BDO dehydrogenase (route ③, illustrated by orange colors). (2*S*,3*S*)-BDO can be synthesized from diacetyl by diacetyl reductase and *S-*specific 2,3-BDO dehydrogenase (route ④, illustrated by blue colors). *ackA* acetate kinase, *adhE* acetaldehyde/alcohol dehydrogenase, *budA* acetolactate decarboxylase, *budB* acetolactate synthase, *budC S-*specific 2,3-BDO dehydrogenase, *dhaD* glycerol dehydrogenase, *ldhA* lactate dehydrogenase, *pfl* pyruvate formate lyase, *pta* phosphate acetyltransferase

2,3-BDO is a natural metabolite which can be produced by different microorganisms such as *Klebsiella oxytoca*, *Bacillus amyloliquefaciens*, and *Enterobacter aerogenes* [92–96]. 2,3-BDO has three stereoisomers: meso-2,3-BDO, (2*R*,3*R*)-BDO, and (2*S*,3*S*)-BDO. The metabolite pathway for 2,3-BDO production is shown in Fig. 6. Two molecules of pyruvate are condensed to one molecule of *S*-acetolactate by acetolactate synthase (*budB*), which is then decarboxylated to *R*-acetoin by acetolactate decarboxylase (*budA*). *R*-Acetoin can be reduced to (2*R*,3*R*)-BDO by (2*R*,3*R*)-BDO dehydrogenase (route ③ of Fig. 6) or to meso-2,3-BDO by meso-2,3-BDO dehydrogenase (*budC*, route ① of Fig. 6). Alternatively, *S*-acetolactate can be converted to diacetyl by non-enzymatic oxidative decarboxylation, which can be further transferred to (2*S*,2*S*)-BDO by (2*S*,3*S*)-BDO dehydrogenase (route ④ of Fig. 6) or to meso-2,3-BDO by meso-2,3-BDO dehydrogenase (route ② of Fig. 6). So far, no new metabolic pathways for 2,3-BDO production have been identified or proposed. Many different stereospecific 2,3-BDO dehydrogenases have been characterized and utilized to construct the desired optically pure 2,3-BDO [97, 98]. It should be mentioned that there may exist different stereospecific 2,3-BDO dehydrogenases in one microorganism, resulting in the accumulation of a mixture of 2,3-BDO stereoisomers. For example, *K. pneumoniae* can accumulate all three 2,3-BDO stereoisomers: meso-2,3-BDO either from *R*-acetoin catalyzed by butanediol dehydrogenase (*budC*) or from *S*-acetoin catalyzed by glycerol dehydrogenase (*gldA*); (2*R*,3*R*)-BDO from *R*-acetoin catalyzed by glycerol dehydrogenase (*gldA*); and (2*S*,3*S*)-BDO from *S*-acetoin catalyzed by butanediol dehydrogenase (*budC*) (Fig. 6) [99]. The conversion of glucose to 2,3-BDO is an oxidation process with a theoretical yield of 1 mol/mol (0.5 g/g glucose). The net NADH generated during the production of 2,3-BDO should be consumed. Thus, (micro)aerobic condition is often applied to 2,3-BDO production. Several wild-type microorganisms can already produce very high titers of 2,3-BDO from different carbon sources. For example, *K. pneumoniae* SDM was reported to be able to accumulate 150 g/L 2,3-BDO with a productivity of 4.21 g/L/h [100]. The main strategies to improve the production of 2,3-BDO can be divided into four categories: (1) reduction of byproduct formation [92, 93]; (2) engineering of cofactor recycling [101]; (3) enhancement of glycolysis and 2,3-BDO synthesis pathway [102]; and (4) heterologous expression of 2,3-BDO synthesis pathway [103–107]. Detailed description of the strategies can refer to several recently published reviews [102, 108–110]. The first two strategies are most efficient for enhancing the production of 2,3-BDO by natural producers. For example, knockout of *ldhA* and *adhE* genes to reduce the accumulation of lactate and ethanol increased the yield of 2,3-BDO to 0.48 g/g in *K. pneumoniae*, which is very close to the maximum theoretical yield [15]. Expression of NADH oxidase to consume excess NADH was also effective to increase the yield of 2,3-BDO [101]. Heterogeneous expression of 2,3-BDO pathways has been tried in different chassis to produce optically pure 2,3-BDO, including *E. coli*, *C. glutamicum*, and *Saccharomyces cerevisiae* [103–107]. Especially, industrially friendly workhorse *S. cerevisiae* was engineered to be able to produce more than 100 g/L enantiopure (2*R*,3*R*)-BDO from a mixture of glucose and galactose, two major carbohydrate components in red algae [106]. Compared to other natural producers, *S. cerevisiae* is more safe and robust in large-scale fermentation, making it a superior host for cost-effective production of 2,3-BDO from renewable resources. Elimination of ethanol and glycerol production and redox rebalancing are the key factors to increase the production of 2,3-BDO by *S. cerevisiae* [103–107]. Production of 2,3-BDO alone or co-production of 1,3-PDO and 2,3-BDO has been demonstrated in pilot scale [111].

### 1,3-Butanediol

1,3-BDO is a non-natural metabolite which has two stereoisomers: *R-*1,3-BDO and *S-*1,3-BDO. Currently, 1,3-BDO is produced as a racemic mixture of *R* and *S* forms by chemical processes [112]. The racemic mixture is commonly used as an organic solvent in food and cosmetic industry. Optically active 1,3-BDO is a very useful building block for antibiotics, pheromones, fragrances, and insecticides [113]. Production of optically pure 1,3-BDO by biological processes has received more and more attention.

**Fig. 7:**
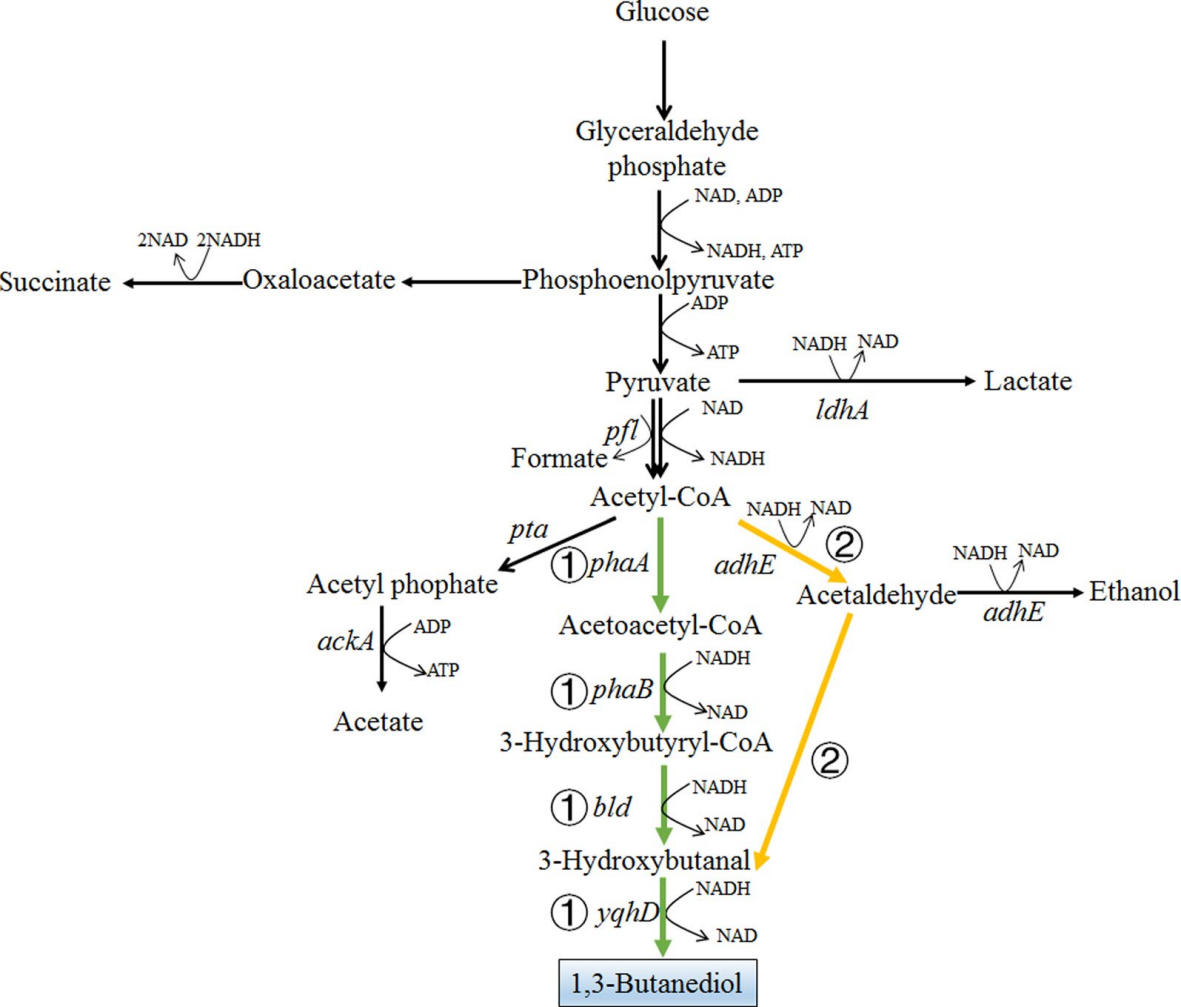
Metabolic pathways for the production of 1,3-butanediol (1,3-BDO). Two metabolic pathways have been proposed to produce 1,3-BDO via acetyl-CoA. In route ① (illustrated by green colors), acetyl-CoA is converted into 1,3-BDO by acetyl-CoA acetyltransferase, acetoacetyl-CoA reductase, butyryl-CoA dehydrogenase, and alcohol dehydrogenase. In route ② (illustrated by orange colors), acetyl-CoA is converted into 1,3-BDO by acetyl-CoA dehydrogenase, aldolase, and alcohol dehydrogenase. *ackA* acetate kinase, *adhE* acetaldehyde/alcohol dehydrogenase, *bld* butyryl-CoA dehydrogenase, *ldhA* lactate dehydrogenase, *pfl* pyruvate formate lyase, *phaA* acetyl-CoA acetyltransferase, *phaB* acetoacetyl-CoA reductase, *pta* phosphate acetyltransferase, *yqhD* NAD(P)H-dependent alcohol dehydrogenases

One non-natural pathway for 1,3-BDO production has been proposed recently (route ① of Fig. 7) [85, 113]. In this pathway, acetyl-CoA is first transferred to acetoacetyl-CoA by acetyl-CoA acetyltransferase, which is further converted to 1,3-BDO by three reduction steps catalyzed by acetoacetyl-CoA reductase, 3-hydroxybutyryl-CoA dehydrogenase, and 1,3-BDO dehydrogenase. No specific enzymes for the last two enzymatic steps have been known. Kataoka et al. tried to introduce *phaA* and *phaB* genes encoding acetyl-CoA acetyltransferase and acetoacetyl-CoA reductase from *Ralstonia eutropha*, and *bld* gene encoding butyryl-CoA dehydrogenase from *Clostridium saccharoperbutylacetonicum* into *E. coli* to catalyze the first three steps [85, 113]. The reduction of 3-hydroxybutanal may be catalyzed by the inherent alcohol dehydrogenases of *E. coli*. The recombinant strain can produce 15.7 g/L *R-*1,3-BDO with 98% ee (enantiomeric excess) and a yield of 0.37 mol/mol glucose (0.18 g/g) in fed-batch fermentation [85, 113]. The yield could be further increased with the reduction of byproduct synthesis and optimization of cofactor regeneration.

Another potential 1,3-BDO synthesis pathway has also been proposed recently. It starts from acetaldehyde, which can be converted to 3-hydroxybutanal by aldolases and aldo–keto reductases (route ② of Fig. 7) [114]. 3-Hydroxybutanal can be reduced to 1,3-BDO by alcohol dehydrogenase. This pathway is shorter than the acetoacetyl-CoA-based pathway, but it is more difficult to be realized. First, there is no efficient aldolase to condense acetaldehyde. Only 2-deoxyribose-5-phosphate aldolase was shown to have very low activity toward acetaldehyde. Second, most of the aldo–keto reductases are non-specific which may also catalyze the reduction of acetaldehyde. Thus, protein engineering should be intensively utilized to improve the activity and specificity of these two enzymes.

Since 1,3-BDO is mainly used as a fine chemical or a solvent in cosmetics, its price is much higher than those of other C2–C4 diols. With the aid of systems metabolic engineering and process engineering, it is very promising to develop an efficient 1,3-BDO bioproduction process for commercial application.

## Summary and future perspectives

C2–C4 diols represent one of the most important categories of bulk chemicals which are classically produced from fossil resource by chemical processes. Due to the depletion of fossil resource and increasing environmental issues, there is an increasing demand for production of these platform chemicals from renewable resources by more sustainable biological processes [2, 115]. Several isomers of C2–C4 diols are non-natural metabolites that lack known metabolic pathways. The recent advancement of synthetic biology and metabolic engineering has enabled us to design new or even non-natural pathways to produce these valuable chemicals [29, 116]. The pathways for all isomers of C2–C4 diols except 1,2-BDO have been proposed and experimentally verified in recent years (Table 1). Similar approaches can also be applied for 1,2-BDO biosynthesis.

**Table 1:** Fermentative production of C2–C4 diols by metabolic engineered strains based on different pathways

Product and strain	Pathways	Genetic modification	Titer (g/L)	Yield (g/g)	Culture, substrate	References
Ethylene glycol						
*Escherichia coli*	d-Xylonate-dependent	∆*xylA,* overexpression of *xdh* and *yqhD*	11.7	0.29	Batch, xylose	[35]
*Escherichia coli*	d-Xylulose 1-phosphate-dependent	∆*xylB*∆*ald,* overexpression of *khk*-*C, aldoB* and *fucO*	20	0.38	Batch, xylose	[39]
*Escherichia coli*	d-Ribulose 1-phosphate-dependent	∆*xylB*∆*ald,* overexpression of *dte, fucK, fucA* and *fucO*	40	0.35	Fed-batch, xylose	[6]
*Corynebacterium glutamicum*	Serine-dependent	∆*sdaA*∆*pabABC,* overexpression of *AsAO, AtSdc, yqhD,* and *serACB*	3.5	0.09	Batch, glucose	[40]
1,3-Propanediol						
*Escherichia coli*	Glycerol-dependent	∆*glpK*∆*gldA*∆*ndh*∆*ptsHI*∆*arcA*, promoter change of *gapA*, overexpression *gpd1, gpp2, dhaBCE, gdrAB, galP, glk, btuR, and yqhD*	135	0.46	Fed-batch, glucose	[61]
*Escherichia coli*	Homoserine-dependent	∆*thrB,* overexpression of *gdh* ^K92V/T195S^, *pdc*, and *yqhD*	0.05	–	Shake flask, glucose	[66]
1,2-Propanediol						
*Escherichia coli*	Methylglyoxal-dependent	∆*zwf*∆*tpiA*∆*adhE*∆*gloA*∆*ldhA*, overexpression *mgsA, gldA, fdh1,* and *fucO*	5.13	0.48	Shake flask, glucose	[10]
*Escherichia coli*	Lactate-dependent	∆*poxB*∆*ackA*-*pta*∆*adhE*∆*dld*∆*ldhA*, overexpression *pct, pdcD,* and *mmsB*	1.04	0.44	Shake flask, glucose	[80]
1,4-Butanediol						
*Escherichia coli*	Succinyl-CoA-dependent	∆*pfl*∆*adhE*∆*mdh*∆*acrA*∆*ldhA*∆*ndh*∆*sad*∆*gabD*∆*puuE*∆*gabT*∆*sucCD*, mutation of *lpdA* ^D354K^, *gltA* ^R163L^, overexpression *of sucD, 4hbd, cat2, ald* and *adh*	99	0.35	Fed-batch, glucose	[22]
*Escherichia coli*	Modified Dahms pathway	∆*xylA*∆*yjhH*∆*yagE*, overexpression *of xylBCDX, kivd* ^V461I^ and *adh*	12	–	Fed-batch, glucose and xylose	[89]
2,3-Butanediol						
*Saccharomyces cerevisiae*	*S-*Acetolactate-dependent	ΔPDCs, overexpression of MTH1, cytoILV2, BsAlsD, and BDH1	100	–	Fed-batch, glucose and galactose	[106]
1,3-Butanediol						
*Escherichia coli*	Acetoacetyl-CoA-dependent	Overexpression of *phaA, phaB,* and *bld*	15.7	0.18	Fed-batch, glucose	[113]

Most of the C2–C4 diols are used as bulk chemicals with relatively low price, and thus the development of efficient biological processes with low production cost is highly important for their commercialization. Since the feedstock often accounts for 25–50% of the total production cost, it is necessary to design different potential routes to utilize various cheap and renewable substrates, especially industrial and agricultural wastes like starch from non-food plants, lignocellulose, raw glycerol, or syngas. Development of integrated processes to convert all components of substrates or industrial waste mixtures to an array of easily separated products to achieve high atom economy offers a promising route to increase the economic viability of biorefinery processes [117, 118]. For example, several recent processes have been developed to produce diols from a mixture of industrial wastes like lignocellulose and raw glycerol, which significantly increase the yield of 1,3-PDO to glycerol due to the extra NADH generated from waste sugars [119]. Co-production of a diol with another value-added, easily separated oxidized product for cofactor recycling can also be applied to increase the process atom economy, as demonstrated in the 1,3-PDO and 3-HP co-production system, 1,3-PDO and lactate co-production system, and 1,3-PDO and glutamate co-production system [53–55]. Direct conversion of CO_2_ with reducing power harnessed from sunlight or electricity by engineering phototrophic organisms or lithoautotrophic microorganisms also provides a potentially sustainable route for large-scale production of cheap diols (e.g., EG) [120].

The development of an efficient cell factory to produce bulk chemicals like C2–C4 diols often requires multiple rounds of the Design–Build–Test–Learn (DBTL) cycle. This is especially important for the production of non-natural metabolites which lack known metabolic pathways and efficient enzymes [31, 34]. In the design stage, a combination of pathway prediction tools with genome-scale metabolic models (GEMs) has been shown to be effective to identify optimal metabolic routes and to enumerate different metabolic engineering targets [18, 121]. However, most of these GEMs only provide stoichiometric constraints without considering kinetic information. The ensemble modeling (EM) approach has been applied to build a genome-scale kinetic model of *E. coli* metabolism satisfying fluxomic data for wild-type and 25 mutant strains under different substrates and growth conditions [122]. A recent effort to integrate big data across multiple levels (fluxomics, metabolomics, transcriptomics, proteomics, and kinetics) into metabolic control analysis (MCA)-based modeling framework, Optimization and Risk Analysis of Complex Living Entities (ORACLE), has allowed the researchers to build a population of large-scale kinetic models to identify key kinetic obstacles for 1,4-BDO production [86]. These attempts have demonstrated the feasibility and potential power of genome-scale kinetic modeling for guiding strain development [123]. In the build stage, multivariate combinatorial pathway engineering to construct pathway libraries through the combinatorial adjustment of biological parts may be quite necessary for the optimization of natural and non-natural heterologous pathways due to the limited predictive power in the design phase [124]. Combinatorial pathway libraries can now be easily built with the fast advancement of DNA synthesis and assembly technology, e.g., Gibson assembly and golden gate. Together with the recently developed genome editing tools such as CRISPR–Cas for gene knockout and knockdown, creation and screening of genomic libraries can be automated by robotics, which can significantly save time and cost for strain development [125]. In the test stage, the recently developed biosensor-based high-throughput screening technologies, microdroplets, and microfluidic chips can significantly accelerate the process of strain screening and characterization [126]. Learning is currently the weakest step in DBTL cycle. Although vast amounts of biological data have been generated, few of them have been systematically analyzed and only very limited information has been extracted. Recently, machine learning and automation techniques have been used to guide strain reengineering, providing a new opportunity to identify and diagnose bottlenecks of bioproduction systems from previously collected data [127].

In summary, the key challenges for biological production of C2–C4 diols lie on how to reduce the production cost. Using cheaper substrates, development of integrated processes, and construction of highly efficient strains should be combined for the development of sustainable diol production systems.

## Abbreviations

DBTL: Design–Build–Test–Learn
EG: ethylene glycol
1,3-PDO: 1,3-propanediol
1,2-PDO: 1,2-propanediol
1,4-BDO: 1,4-butanediol
2,3-BDO: 2,3-butanediol
1,3-BDO: 1,3-butanediol
1,2-BDO: 1,2-butanediol
PET: polyethylene terephthalate
PTT: polypropylene terephthalate
PBT: polybutylene terephthalate
PEP: phosphoenolpyruvate
PTS: phosphotransferase system
